# Mitigation pilot contamination based on matching technique for uplink cell-free massive MIMO systems

**DOI:** 10.1038/s41598-022-21241-0

**Published:** 2022-10-07

**Authors:** Abdulrahman Al Ayidh, Yusuf Sambo, Muhammad Ali Imran

**Affiliations:** 1 School of Engineering, University of Glasgow, Glasgow, UK; 2 College of Engineering, King Khalid University, Abha, Kingdom of Saudi Arabia

**Keywords:** Engineering, Electrical and electronic engineering

## Abstract

In this paper, the cell-free massive multiple input multiple output (MIMO) network is affected by the pilot contamination phenomenon when a large number of users and a small number of available pilots exists, the quality of service (QoS) will deteriorate due to the low accuracy of the channel estimation because some of users will use the same pilot. Therefore, we address this problem by presenting two novel schemes of pilot assignment and pilot power control design based on the matching technique for the uplink of cell-free massive MIMO systems to maximize spectral efficiency. We first formulate an assignment optimization problem in order to find the best possible pilot sequence to be used by utilizing genetic algorithm (GA) and then propose a Hungarian matching algorithm to solve this formulated problem. Regarding the power control design, we formulate a minimum-weighted assignment problem to assign pilot power control coefficients to the estimated channel’s minimum mean-squared error by considering the access point (AP) selection. Then, we also propose the Hungarian algorithm to solve this problem. Simulation results show that our proposed schemes outperform the state-of-the-art techniques concerning both the pilot assignment and the pilot power control design by achieving a 15% improvement in the spectral efficiency. Finally, the computational complexity analysis is provided for the proposed schemes compared with the state-of-the-art techniques.

## Introduction

The future generations of wireless networks will lay the foundation for all aspects of life, society, and industry due to their high QoS regarding ultra-high reliability and ultra-low latency . For example, virtual and augmented reality, e-Health and the Internet of Things (IoTs) will all be possible thanks to these future networks^1,2^. Cell-free massive MIMO network is a recently-emerging concept for deploying massive MIMO systems without the restriction of cells^3^. Therefore, the cell-free massive MIMO allows user equipments (UEs) to be served by all base stations (BSs) simultaneously across a large coverage area instead of only single BS. In other words, a large number of BSs, also known as APs, are randomly distributed and connected to the Central Processing Unit (CPU) via fronthaul links to coordinate data transmission at the same time-frequency resources using spatial multiplexing techniques^3–5^. In terms of uplink and downlink achievable data rates, the performance of the cell-free massive MIMO systems is compared to the small-cell massive MIMO systems^4,6,7^. It has been discovered that the cell-free massive MIMO systems outperform fully distributed small-cell systems in terms of $95\%$-likely per-user throughput. Moreover, by utilizing max-min power control, the cell-free systems can provide uniformly good service to all UEs within the service area.

The process of channel estimation is considered to be one of the most essential operations in the cellular and cell-free massive MIMO systems, as it directly influences the computations of precoding and detection vectors which are utilized for the uplink and downlink data transmission^8,9^. Regarding the time division duplexing (TDD) communication protocol, recent studies have developed pilot-based channel estimate algorithms in which UEs communicate $\tau$-length pilot sequences to APs. The channel coherence time and the number of UEs are related to each other in the channel estimation process^2^. Furthermore, the pilot sequences assigned to UEs might be orthogonal or non-orthogonal, for instance, orthogonal pilot sequences can be allocated when there is a high $\tau _{c}$ coherence interval and a limited number of UEs. However, when $\tau _{c}$ is minimal, it is preferable to utilize non-orthogonal pilot sequences to reduce the resources required for channel estimation^9^. Therefore, the interference between the transmitted pilot signal from the desired UE and other transmitted pilot signals from other UEs at each AP leads to the degradation of the estimated channel accuracy, impacting system performance. The term for this issue is called pilot contamination. Accordingly, pilot assignment techniques and power control design approach can be used to mitigate the pilot contamination effect on the system performance^2,4,9,10^.

Regarding the pilot power control design, during the training phase, if all pilot signals are transmitted at full power, a UE with a weak channel might be highly contaminated by UEs with strong channels. As a result, the total system performance deteriorates. In order to handle this issue, the authors in^9^ proposed that pilot power coefficients be designed to increase the channel estimation accuracy during the training phase. They presented a min-max optimization problem that aims to reduce the largest of all UEs’ normalized mean-squared channel estimation errors, and this proposed optimization problem is non-convex. Then, they changed it to the second-order Taylor approximation. However, the proposed scheme in^9^ has higher computational complexity in the real-time implementation when the cell-free network has large number of both APs and UEs. On the another side, the performance of the cell-free massive MIMO systems has been evaluated in^4,11–16^ using the pilot assignment technique to mitigate the pilot contamination effect and enhance the quality of the channel estimation. The authors of^4^ proposed random and greedy pilot assignment schemes. Particularly, the random scheme is a simple algorithm of the pilot assignment and aims to allocate available $\tau$ pilots to *K* UEs, and then the reusable pilot sequences are assigned to the remaining $K - \tau$ UEs without considering the influence on the system performance. In addition, they proposed the greedy scheme, which is performed iteratively to maximize the minimum data rate of the UE. Unfortunately, this scheme cannot provide the optimal pilot assignment in the cell-free massive MIMO systems since it is limited to the local optimum. A Tabu search-based pilot assignment strategy with low complexity is proposed in^11^ to repeatedly search for the sub-optimal pilot assignment. Another direction for the pilot assignment based on graph theory has been proposed in^12,16^. A graph colouring-based and weighted graph-based pilot assignment schemes are presented where a limited number of neighboring APs serve each UE. Consequently, depending on the APs selection algorithm, the large-scale fading coefficients between APs and UEs are utilised to generate an interference graph. Therefore, the optimal pilot assignment is determined by modifying the generated interference graph. To increase the average downlink achievable rate, Dang et al.^14^ proposed a pilot assignment technique based on the genetic algorithm (GA). The GA-based approach outperforms other traditional schemes, according to numerical results. However, the GA lacks local search-ability and is sensitive to ”rapid” convergence.

Motivated by the mentioned considerations previously, this work aims to mitigate the pilot contamination effect on the performance of the cell-free massive MIMO systems by proposing two schemes for the pilot assignment and the pilot power control design. The contributions of this work are as follows

- The iterative Hungarian scheme is proposed to solve the formulated assignment optimization problem in order to obtain better-selected pilot sequences. The reason for doing that is to reduce the complexity of the GA by using the selected pilot sequences as input (*termed populations*) instead of putting $\tau ^{K}$ possible combinations of the pilot sequences in the conventional GA. Based on this, it can be guaranteed that the GA does not lack local-search ability.
- We also propose a lower complexity pilot power control design for the uplink cell-free massive MIMO systems based on matching theory. We formulate a minimum weighted assignment optimization problem and use the Hungarian algorithm in order to obtain the optimal assignment between the pilot power control coefficients and the minimum channel estimation error for all UEs.
- Comprehensive simulation results are provided to demonstrate the performance of the proposed pilot assignment and pilot power control strategies under an extensive set of the cell-free massive MIMO scenarios. In particular, the number of APs, the number of antennas, the number of available pilot $\tau$, and the number of UEs in the network have been analyzed in terms of the total uplink net throughput. In addition, the computational complexity analysis for the proposed schemes is studied in this work.

## System model

In this paper, the uplink cell-free massive MIMO systems is considered where the communication between *M* APs and *K* single-antenna UEs, randomly distributed in the coverage area, is coordinated by a CPU. Each AP is equipped with $N_{r}$ receive antennas. Also, each AP has the option of being activated or deactivated in order to reduce the requirements for backhaul connection. The sets $\delta ^{\text {A}} = \{m_{1}^{\text {A}},\ldots ,m_{M_{\text {Activate}}}^{\text {A}}\}$ and $\delta ^{\text {D}} = \{m_{1}^{\text {D}},\ldots ,m_{M_{\text {Deactivate}}}^{\text {D}}\}$ denote the sets of activated and deactivated APs, respectively, such that $|\delta ^A| + |\delta ^D| = M$. The AP activation can be done by utilizing the largest-large-scale-fading scheme^10^. In addition, it is assumed that each UE in the network is served by a subset of $\delta ^{\text {A}}$ . TDD is utilized in this work to process the transmission from *K* UEs to *M* APs.

Furthermore, by leveraging the estimated channels at *M* APs, the transmitted signals from *K* UEs in the coverage area can be decoded. Let $g_{k,m} \in {\mathbb {C}}^{N_{r}\times 1}$ denote the channel coefficient vector between $k_\text {th}$ UE and $m_\text {th}$ AP and it is expressed as

1 $$\begin{aligned} g_{k,m} = \sqrt{\beta _{k,m}} h_{k,m}, \end{aligned}$$

where $\beta _{k,m}$ represents the large scale fading and $h_{k,m} \in {\mathbb {C}}^{N_{r}\times 1}$ denotes the small scale fading vector. Each UE and AP is likely to have a various set of scatters due to the random distribution of APs and UEs over a wide service area. Thus, $\{h_{k,m}\}$, $k=1,\ldots ,K$ and $m=1,\ldots ,M$, are assumed to be independent identically distributed (i.i.d.) ${\mathscr {C}}{\mathscr {N}} (0,1)$ random variables (RVs). Is is also assumed that all APs are connected to the CPU via fronthaul links to serve all *K* UEs at the same time.

### Uplink channel estimation

The parameter $\tau _{c}$ denotes the length of coherence interval (in symbols), which is larger than the length of the uplink training phase $\tau$ (in symbols). The pilot sequence for $k_\text {th}$ UE is given by $\sqrt{\tau \eta _{k}} \phi _{k} \in {\mathbb {C}}^{\tau \times 1}$ where $||\phi _{k}||^{2} = 1$ and $\eta _{k}$ is the power control coefficient for $k_\text {th}$ UE, where $0<\eta _{k}\le 1$. Then, $m_\text {th}$ AP receives the $\tau \times 1$ pilot vector from all UEs to be used for the channel estimation and this vector is expressed as

2 $$\begin{aligned} Y_{p,m} = \sqrt{\tau \rho _{p}} \sum _{k=1}^{K} g_{k,m} \eta _{k}^{\frac{1}{2}} \phi _{k}^{H} + {\mathbf {N_{p,m}}}, \end{aligned}$$

where $\rho _{p}$ denotes the normalized signal-to-noise ratio (SNR) of each pilot symbol (normalized by the noise power), where the noise power is $-174 \frac{{\text {dBm}}}{{\text {Hz}}} + 10 \log _{10} (B) +{ \text {Noise Figure}}$, where *B* is the system bandwidth. ${\mathbf {N_{p,m}}}$ gives the matrix of additive noise at $m_\text {th}$ AP with size $N_{r}\times \tau$. Also, all entire elements of ${\mathbf {N_{p,m}}}$ are assumed to be i.i.d. ${\mathscr {C}}{\mathscr {N}} (0,1)$ RVs. The minimum mean squared-error (MMSE) technique is utilized to estimate the channel ${g}_{k,m}$ between $k_\text {th}$ UE and $m_\text {th}$ AP after performing projection the received pilot signal $Y_{p,m}$ onto $\phi _{k}$:

3 $$\begin{aligned} \begin{aligned} \hat{y}_{p,k,m}&= \phi _{k}^{H} Y_{p,m}\\&= \sqrt{\tau \rho _{p}} g_{k,m} \eta _{k}^{\frac{1}{2}} + \sqrt{\tau \rho _{p}}\sum _{k^{'} \ne k}^{K} g_{m, k^{'}} \eta _{k^{'}}^{\frac{1}{2}} \phi _{k}^{H} \phi _{k^{'}} + \phi _{k}^{H} {\mathbf {N_{p,m}}}.\\ \end{aligned} \end{aligned}$$

Thus, the MMSE estimate of the channel between $k_\text {th}$ UE and $m_\text {th}$ AP is given as

4 $$\begin{aligned} \hat{g}_{k,m} = c_{k,m} \left( \sqrt{\tau \rho _{p}} g_{k,m} \eta _{k}^{\frac{1}{2}} +\sqrt{\tau \rho _{p}} \sum _{k^{'} \ne k}^{K} g_{m, k^{'}} \eta _{k^{'}}^{\frac{1}{2}} \phi _{k}^{H} \phi _{k^{'}} + \phi _{k}^{H} {\mathbf {N_{p,m}}}\right) , \end{aligned}$$

where $c_{k,m} = \frac{\sqrt{\tau \rho _{p}} \beta _{k,m} \eta _{k}^{\frac{1}{2}}}{\tau \rho _{p} \sum _{k^{'} = 1}^{K} \beta _{k^{'},m} \eta _{k^{'}} |\phi _{k}^{H} \phi _{k^{'}}|^{2} + 1 }$. In addition, each AP in the coverage area individually estimates the channel and there is no cooperation among APs on the channel estimation process.

### Uplink data transmission

The transmitted signal from $k_\text {th}$ UE is denoted by $x_{k} = \sqrt{\eta _{k}}s_{k}$, where $s_{k}$, that satisfies ${\mathbb {E}}\{ |s_{k}|^{2}\}=1$, represents $k_\text {th}$ UE transmitted symbol. The received signal from all UEs in the cell-free network to $m_\text {th}$ AP is given by

5 $$\begin{aligned} y_{u,m} = \sqrt{\rho _{u}} \sum _{k=1}^{K} g_{k,m} x_{k} + n_{u,m}, \end{aligned}$$

where $n_{u,m} \in {\mathbb {C}}^{N_{r}\times 1}$ presents the noise at $m_\text {th}$ AP where its elements are assumed to be i.i.d. ${\mathscr {C}}{\mathscr {N}} (0,1)$ RVs and $\rho _{u}$ is the uplink normalized SNR data transmission (also normalized by the noise power, as mentioned in the previous section).

In order to detect the transmitted symbol from $k_\text {th}$ UE, the AP sends the product of its $y_{u,m}$ received signal, from *K* UEs using the obtained estimated channel $\hat{g}_{k,m}$, to the CPU via the fronthaul link^9^. Thus, the CPU receives

6 $$\begin{aligned} r_{u,k} = \sum _{m \in \delta ^{\text {A}}} \sum _{n=1}^{N_{r}} \left[ \hat{g}_{k,m} \right] _{n}^{*} \left[ y_{u,m} \right] _{n}. \end{aligned}$$

### Spectral efficiency

Analysis techniques that are similar to those used in^4,9^, are utilized in this subsection to obtain the derivation of the uplink spectral efficiency (SE). Therefore, the main function of the CPU in the cell-free MIMO network is to detect the desired signal $x_{k}$ from $r_{u,k}$. In addition, similar assumption is considered in this paper to perform the detection of $x_{k}$, the statistical knowledge of the channel that is only used by the CPU. Thus, the received signal at the CPU from $k_\text {th}$ UE as shown in (6) is decomposed as follows

7 $$\begin{aligned} r_{u,k} = \textsc {DS}_{k} \cdot x_{k} + \textsc {BU}_{k} \cdot x_{k} + \sum _{k^{'} \ne k}^{K} \textsc {UI}_{kk^{'}} \cdot x_{k} + \textsc {N}_{k}, \end{aligned}$$

where

$$\begin{aligned}{}&\textsc {DS}_{k} \triangleq \sqrt{\rho _{u}} \ {\mathbb {E}}\left\{ \sum _{m \in \delta ^{\text {A}}} \sum _{n=1}^{N_{r}} [\hat{g}_{mk}]_{n}^{*} [g_{mk}]_{n}\right\} , \\&\textsc {BU}_{k} \triangleq \sqrt{\rho _{u}} \sum _{m \in \delta ^{\text {A}}} \sum _{n=1}^{N_r} [\hat{g}_{mk}]_{n}^{*} [g_{mk}]_{n} - \textsc {DS}_{k}, \\&\textsc {UI}_{kk^{'}} \triangleq \sqrt{\rho _{u}} \sum _{m \in \delta ^{\text {A}}} \sum _{n=1}^{N_r} [\hat{g}_{mk}]_{n}^{*} [g_{mk^{'}}]_{n}, \end{aligned}$$

and

$$\begin{aligned} \textsc {N}_{k} \triangleq \sum _{m \in \delta ^{\text {A}}} \sum _{n=1}^{N_r} [\hat{g}_{mk}]_{n}^{*} [n_{u,m}]_{n}, \end{aligned}$$

present the desired signal, the beamforming gain uncertainty, and the interference caused by $k^{'}_{\text {th}}$ UE. Thus, the uplink SE is obtained by considering the second, third and fourth term as an effective noise as well as using the worst case Gaussian noise argument^4,9,10,17^, as follows

8 $$\begin{aligned} \text {SE} = \frac{1-\tau /\tau _{c}}{2} \sum _{k=1}^{K}\log _{2} (1 + \text {SINR}_{k}), \end{aligned}$$

where $\text {SINR}_{k}$ denotes signal-to-interference-plus-noise ratio which is written as

9 $$\begin{aligned} \text {SINR}_{k} = \frac{ \begin{vmatrix} \textsc {DS}_{k} \end{vmatrix}^{2}}{{\mathbb {E}} \Big \{ \begin{vmatrix}\textsc {BU}_{k} \end{vmatrix}^{2} \Big \} + {\mathbb {E}} \Big \{ \begin{vmatrix} \textsc {UI}_{kk^{'}} \end{vmatrix}^{2} \Big \} + {\mathbb {E}} \Big \{ \begin{vmatrix} \textsc {N}_{k} \end{vmatrix}^{2} \Big \}}. \end{aligned}$$

Finally, the closed form expression of (9) can be obtained by following equations as

10 $$\begin{aligned} \textsc {DS}_{k} = N_{r} \sqrt{\rho _{u} \eta _{k}} \sum _{m\in \delta ^{\text {A}}} \sqrt{\tau \rho _{p}} \beta _{k,m} c_{k,m}, \end{aligned}$$

11 $$\begin{aligned} {\mathbb {E}} \{ |\textsc {BU}_{k}|^{2}\} = N_{r} \rho _{u} \eta _{k} \sum _{m\in \delta ^{\text {A}}} \sqrt{\tau \rho _{p}} \beta _{k,m}^{2} c_{k,m}, \end{aligned}$$

and

12 $$\begin{aligned} \begin{aligned} {\mathbb {E}} \{ |\textsc {UI}_{kk^{'}}|^{2}\}&= N_{r}^{2} \rho _{u} \eta _{k^{'}} |\phi _{k}^{H} \phi _{k^{'}}|^{2} (\sum _{m \in \delta ^{\text {A}}} \sqrt{\tau \rho _{p}} \beta _{k,m} c_{k,m} \frac{\beta _{m,k^{'}}}{\beta _{k,m}})^{2}\\&\quad + N_{r} \rho _{u} \eta _{k^{'}} \sum _{m \in \delta ^{\text {A}}}\sqrt{\tau \rho _{p}} \beta _{k,m} \beta _{m,k^{'}} c_{k,m}.\\ \end{aligned} \end{aligned}$$

Thus, (10), (11) and (12) are used to obtain (9).

## Mitigation pilot contamination methodology

When numerous UEs communicate with the same AP, the pilot contamination phenomenon occurs when the UEs utilize the same pilot sequence. As a consequence, these UEs are assigned the same pilot sequence, resulting in a reduction in the accuracy of the estimated channels. As a result, the effect of pilot contamination is addressed in this paper by proposing two schemes of the pilot assignment and the pilot power control.

### Pilot assignment scheme

The pilot set $\Delta _{ \phi }$ includes $\{1,2,\ldots ,\tau \}$ orthogonal pilot sequences and is given as

13 $$\begin{aligned} \Delta _{ \phi } = \{\phi _{1}, \phi _{2},\ldots ,\phi _{\tau } \}. \end{aligned}$$

*K* pilot sequences are randomly selected from $\Delta _{ \phi }$ and then these selected pilot sequences are assigned to *K* UEs. Therefore, the main aim of this stage in this paper is to maximize the uplink SE by finding the optimal assignment between the pilot sequences to *K* UEs in order to alleviate the effect of the pilot contamination phenomenon. Thus, the optimization problem is formulated as

14 $$\begin{aligned} \begin{aligned} \max _{{\mathscr {J}}_{l}} \quad&\left( \frac{1-\tau /\tau _{c}}{2} \sum _{k=1}^{K}\log _{2} (1 + \text {SINR}_{k})\right) ,\\ \end{aligned} \end{aligned}$$

where ${\mathscr {J}}_{l}$ gives all possible cases of the pilot assignment in which ${\mathscr {J}}_{l} = \{\phi _{l}^{1},\phi _{l}^{2},\ldots ,\phi _{l}^{K}\}$ and $l = \{1,2,\ldots ,\tau ^{K}$}. Thus, $\phi _{l}^{k}$ is selected from $\Delta _{ \phi }$ and then assigned to $k_\text {th}$ UE. The optimization problem in (14) is NP-hard^12^. The exhaustive searching technique can solve this problem, but this scheme suffers from huge computational complexity, especially when there is a large number of UEs in the coverage area. Therefore, we propose a novel pilot assignment scheme based on an iterative Hungarian strategy and the GA by solving the pilot assignment optimization problem in (14) for the uplink cell-free massive MIMO systems. Moreover, the iterative Hungarian scheme is utilized to obtain the best populations of the GA instead of using $\tau ^{K}$ populations as an input. Then, the GA is used to find the optimal pilot sequence for each UE in the coverage area.

#### The iterative Hungarian technique

It is assumed that there are ${\mathscr {J}}_{l}$ possible cases of pilot assignment when $l = 1,2,\ldots ,K$ rather than $l = 1,2,\ldots ,\tau ^{K}$. Then, ${\mathscr {J}}_{l}$ is randomly generated and the spectral efficiency for each UE is calculated based on the entire elements of ${\mathscr {J}}_{l}$ in order to produce the reward matrix as shown in the example of one iteration in Table 1.

**Table 1:** An example of the reward matrix between ${\mathscr {J}}_{l}$ and *K* UEs for $K = l = 4$.

	$UE_1$	$UE_2$	$UE_3$	$UE_4$
${\mathscr {J}}_{1}$	$\text {SE}_{1,1}(\varvec{{\phi _{1}^{1}}})$	$\text {SE}_{1,2}(\varvec{{\phi _{1}^{2}}})$	$\text {SE}_{1,3}(\varvec{{\phi _{1}^{3}}})$	$\text {SE}_{1,4}(\varvec{{\phi _{1}^{4}}})$
${\mathscr {J}}_{2}$	$\text {SE}_{2,1}(\varvec{{\phi _{2}^{1}}})$	$\text {SE}_{2,2}(\varvec{{\phi _{2}^{2}}})$	$\text {SE}_{2,3}(\varvec{{\phi _{2}^{3}}})$	$\text {SE}_{2,4}(\varvec{{\phi _{2}^{4}}})$
${\mathscr {J}}_{3}$	$\text {SE}_{3,1}(\varvec{{\phi _{3}^{1}}})$	$\text {SE}_{3,2}(\varvec{{\phi _{3}^{2}}})$	$\text {SE}_{3,3}(\varvec{{\phi _{3}^{3}}})$	$\text {SE}_{3,4}(\varvec{{\phi _{3}^{4}}})$
${\mathscr {J}}_{4}$	$\text {SE}_{4,1}(\varvec{{\phi _{4}^{1}}})$	$\text {SE}_{4,2}(\varvec{{\phi _{4}^{2}}})$	$\text {SE}_{4,3}(\varvec{{\phi _{4}^{3}}})$	$\text {SE}_{4,4}(\varvec{{\phi _{4}^{4}}})$

**Figure 1:**
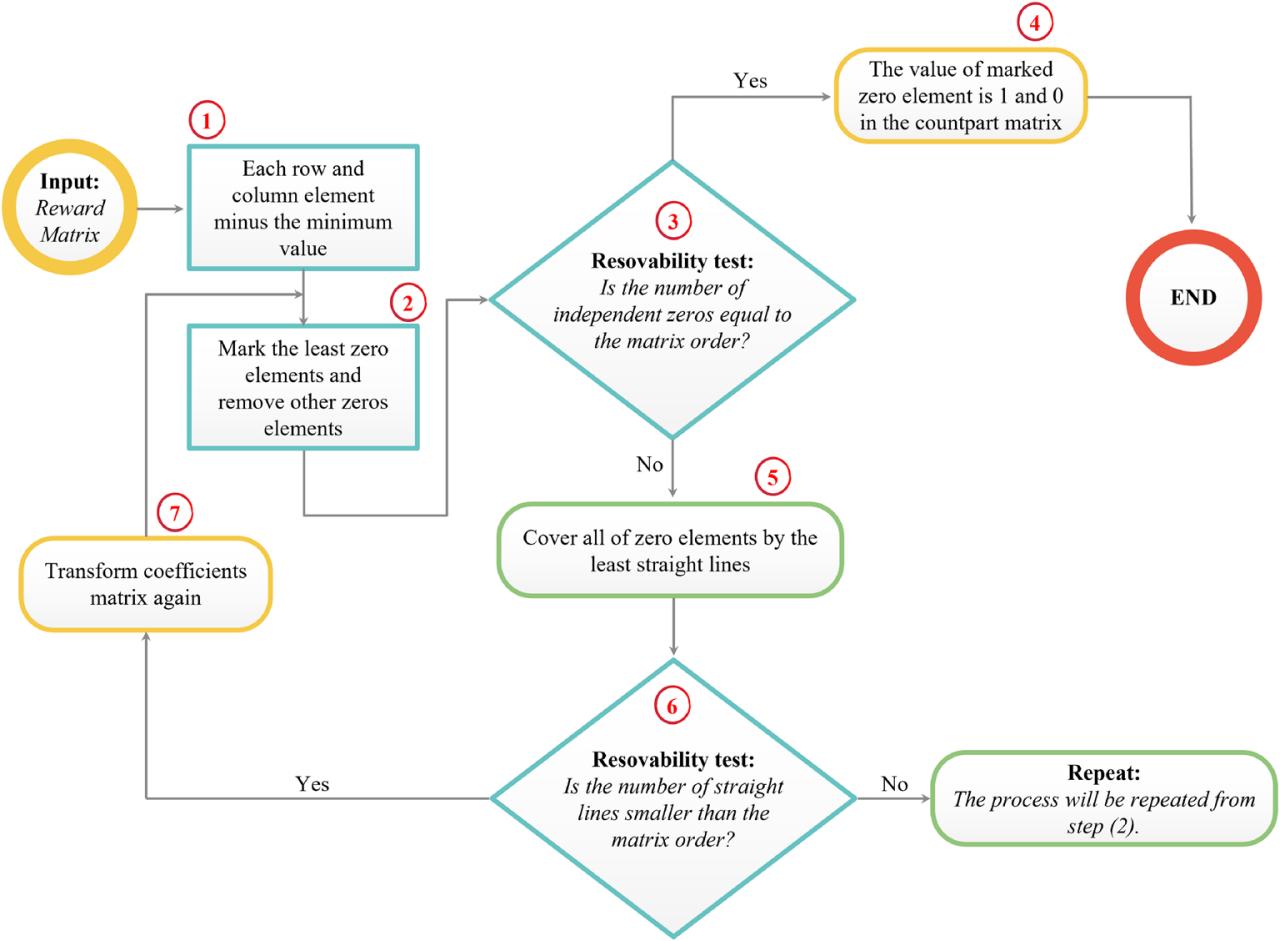
Flow chart of the Hungarian algorithm.

Therefore, an assignment optimization problem is formulated as

15 $$\begin{aligned} \begin{aligned} \max _{\alpha _{l,k} \in [0,1]} \quad&\sum _{l=1}^{K} \sum _{k=1}^{K} \text {SE}_{l,k} (\alpha _{l,k}),\\ \text {s.t.} \quad&\sum _{l=1}^{K} \alpha _{l,k} = 1, \ \forall k, \\ \quad&\sum _{k=1}^{K} \alpha _{l,k} = 1, \text {for} \ \forall l, \\ \end{aligned} \end{aligned}$$

where $\text {SE}_{l,k}$ is the $k_{\text {th}}$ spectral efficiency corresponding to $l_{\text {th}}$ pilot sequence, and $\alpha _{l,k}$ indicates $l_{th}$ pilot sequence ($\phi _{l}^{k}$) is assigned to $k_\text {th}$ UE. The constrains of the optimization problem are to ensure $l_{\text {th}}$ pilot sequence is assigned to only one UE. Furthermore, problem (15) can be solved by applying the Hungarian algorithm, as described in Algorithm 1. This algorithm is iteratively performed *K* times in order to prepare the *K* possible populations for the GA in the following section. Algorithm 1 summarises the whole procedures of the proposed iterative Hungarian scheme. The first step is used to obtain $\tau$ orthogonal sequences based obtaining the right singular value decomposition. Then, it is required to initialize ($\epsilon$) random number from the range $[1,\tau ]$ and its corresponding orthogonal pilot sequence can be determined from the previous step. Once ${\mathscr {J}}_{l} = [\phi _{l}^{1},\phi _{l}^{2},\ldots ,\phi _{l}^{K}]$ is obtained, it is required to compute its corresponding $[\text {SE}_{l,1}(\phi _{l}^{1}),\text {SE}_{ l,2}(\phi _{l}^{2}),\ldots ,\text {SE}_{l,K}(\phi _{l}^{K})]$ by using $\text {SE}_{l,k}(\phi _{l}^{k}) = \frac{1-\tau /\tau _{c}}{2} \log _{2} (1 + \text {SINR}_{l,k})$. The reward matrix is generated as illustrated in Table 1 for each $i_{\text {th}}$ iteration. Accordingly, the Hungarian algorithm is used to solve the assignment optimization problem in (15). Moreover, as shown in Fig. 1, the suggested algorithm’s high-level diagram begins by assigning $UE_{k}$ to ${\mathscr {J}}_{l}$ randomly. Then, the Hungarian algorithm starts by reducing each row in the input reward matrix, which consists of the computed $\text {SE}_{l,k}$ with all ${\mathscr {J}}_{l}$, by subtracting the minimum item in each row from all other items in the same row, and then repeating the process for each column. Then, we look for the convenient ${\mathscr {J}}_{l}$ for each $UE_{k}$. If $UE_{k}$ is already assigned to ${\mathscr {J}}_{l}$, it is better to be assigned with another ${\mathscr {J}}_{l}$, we prime the alternative before moving on to the next ${\mathscr {J}}_{l}$ candidate pilot sequences; however, if that is the only ${\mathscr {J}}_{l}$ pilot sequences for which $UE_{k}$ is qualified, we would like to reassign any other $UE_{k}$ to those ${\mathscr {J}}_{l}$, this step is known as a percolation process. We reassign $UE_{K}$ to their ${\mathscr {J}}_{K}$ to guarantee the assignment can provide the maximum SE, which is the resolvability test. As a result, we can be confident that we make progress toward our goal of identifying the best assignment with each iteration in order to prepare $K \times K$ the best possible populations.

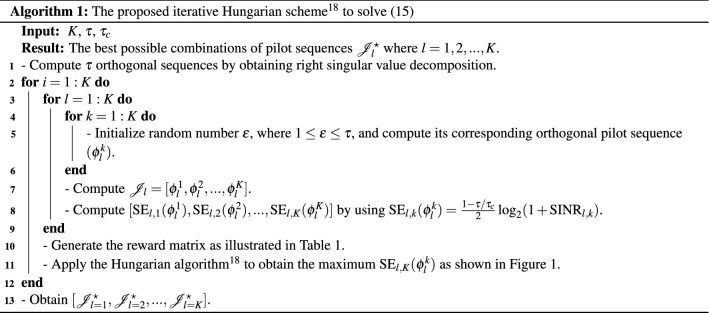



#### The proposed GA scheme

GA is an efficient stochastic method for solving optimal problems that is based on natural selection and natural genetics^19–21^. The GA technique is used to solve the optimization problem, and it contains population initialization, fitness value evaluation, and genetic operations including selection, crossover, and mutation to produce the next generation population. The GA operations are iteratively repeated until the best solution is achieved^21^. The main difference between utilizing the conventional and proposed GA is using the Hungarian algorithm to select the best populations to be inserted in the GA. This will reduce the computational complexity of the pilot assignment, especially when many UEs exist in the cell-free network.

Algorithm 2 shows the main steps of the proposed GA to obtain the optimal pilot sequences to be assigned to *K* UEs in the uplink cell-free massive MIMO systems. The possible pilot sequences are obtained by using the iterative Hungarian algorithm, and these possible pilot sequences are considered as an input of the GA and termed as *populations* which includes multiple chromosomes. Thus, *Populations* can be given as

16 $$\begin{aligned} \textit{Populations} = \left[ {\mathscr {J}}_{l=1}^{\star },{\mathscr {J}}_{l=2}^{\star },\ldots ,{\mathscr {J}}_{l=K}^{\star }\right] . \end{aligned}$$

Then, each chromosome is expressed as ${\mathscr {J}}_{l=k}^{\star } = [\phi _{k}^{1},\phi _{k}^{2},\ldots ,\phi _{k}^{K}]$. The chromosome includes multiple genes. These genes are encoded to $\tau$ integer numbers, such that $\{1,2,\ldots ,\tau \}$. Therefore, this process is called *gene encoding*. After that, each chromosome has its fitness value which is the total SE for the uplink cell-free massive MIMO systems and this fitness value can be obtained by utilizing Eq. (8). This process called *fitness evaluation*. Inside each GA iteration, there are four main steps. **Step (1)** is the selection process. This process is to select parents to perform crossover process and these selected parents can be obtained by Roulette Wheel Selection technique. When ball is thrown in, each chromosome with higher fitness value has a chance to be selected. **Step (2)** is to obtain new offspring by using partially-matched crossover (PMX) crossover technique because it is not permissible to highly repeat genes on the new offspring^22^. **Step (3)** describes the mutation process in order to avoid local optimum^19,22^. This process can be done by choosing random gene with the GA mutation probability and swapping the selected random gene by randomly choosing another gene from the range $[1,\tau ]$. Then, the updated new offspring is evaluated by using Eq. (8) and the populations are updated by the new offspring after the crossover and mutation operations. All of the previous steps are repeated if $l < \Gamma$ iterations. Finally, *K* pilot sequences with the highest fitness value in the last population are obtained to be assigned to *K* UEs.
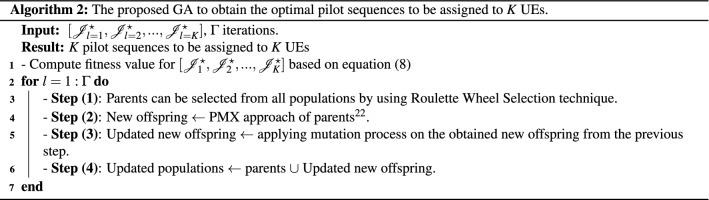


### Pilot power control design

We propose a lower complexity pilot power control design for the uplink cell-free massive MIMO systems based on matching theory. It has been formulated as a minimum weighted assignment optimization problem in order to assign the pilot power control coefficient to the minimum channel estimation error for each UE. The formulated assignment problem is expressed as

17 $$\begin{aligned} \begin{aligned} \max _{\alpha _{i,k} \in [0,1]} \quad&\sum _{i=1}^{K} \sum _{k=1}^{K} \left( \sum _{m\in \delta ^{\text {A}}} (1 - \sqrt{\tau \rho _{p}} \eta _{i,k}^{\text {1/2}} c_{k,m})\right) (\alpha _{i,k}),\\ \text {s.t.} \quad&\sum _{i=1}^{K} \alpha _{i,k} = 1, \ \forall k, \\ \quad&\sum _{k=1}^{K} \alpha _{i,k} = 1, \text {for} \ \forall i. \\ \end{aligned} \end{aligned}$$

**Figure 2:**
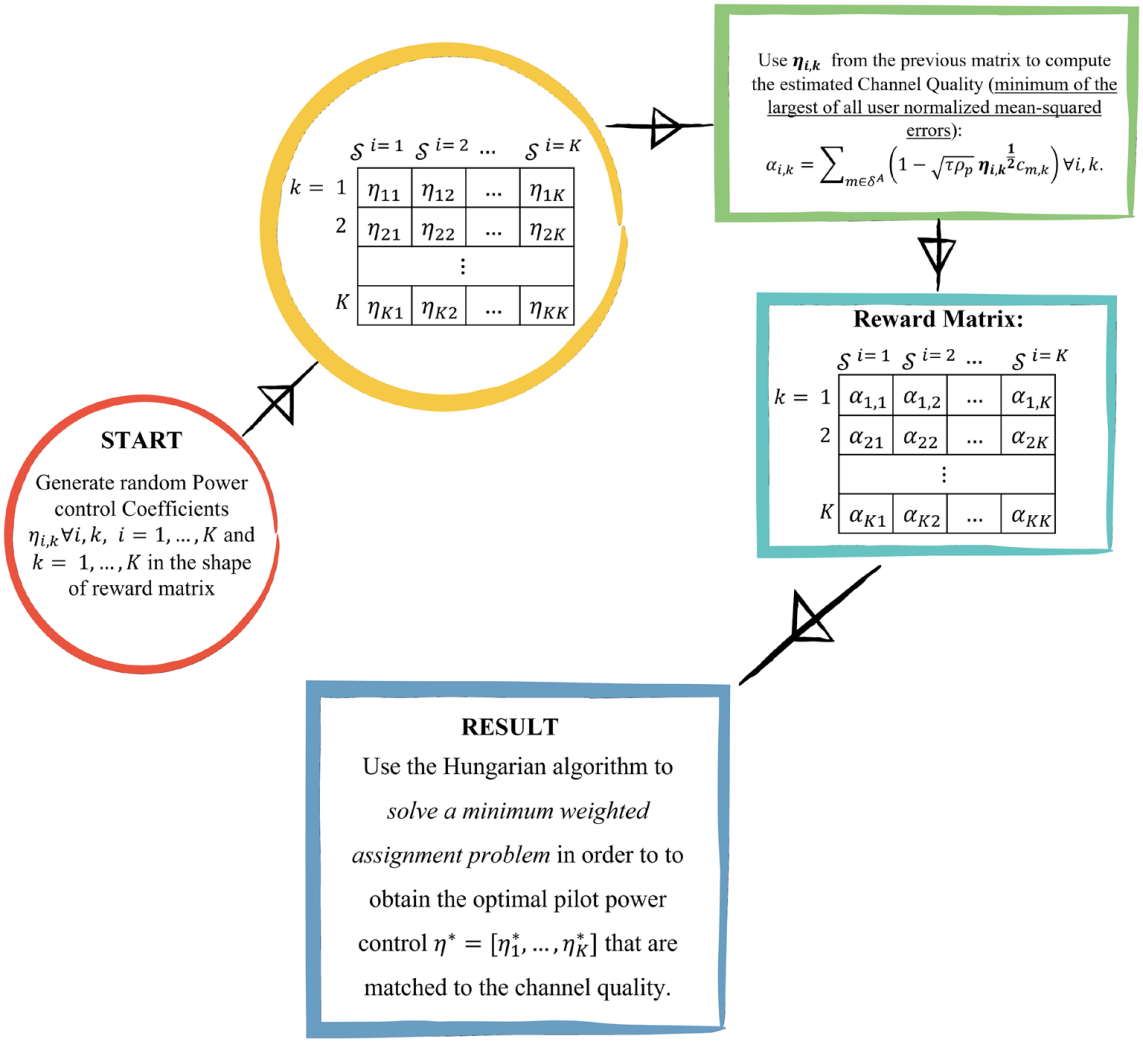
Steps of the proposed pilot power control design based on matching technique to enhance the uplink cell-free massive MIMO systems performance.

The previous formulated assignment problem in this work does not involve some continuous variables $\eta _{k}$ as^9^, because it has been assumed that $\eta _{i,k}$ is a fixed random variable of the pilot power control coefficient, such that $0 \le \eta _{i,k} \le 1$. Therefore, the reward matrix of the proposed matching scheme in this section includes the estimated channel quality based on random values of all UEs pilot power coefficients. Figure 2 demonstrates the steps to obtain the optimal pilot power control coefficients for all UEs in the cell-free network. Firstly, it is generated *K* random pilot power $\eta _{i,k}$ for each UE, where $i = {1,2,\ldots ,K}$ that indicates the number of random pilot power. Secondly, each $\eta _{i,k}$ is substituted in $\left( \sum _{m\in \delta ^{\text {A}}}\left( 1 - \sqrt{\tau \rho _{p}} \eta _{i,k}^{\text {1/2}} c_{k,m}\right) \right)$ in order to obtain the estimated channel quality. Therefore, the reward matrix with size $K \times K$ is obtained based on the values of the estimated channel quality corresponding to the random pilot power for each UE. Finally, the Hungarian algorithm is utilized to find the minimum of the largest of all UE normalized mean-squared errors with optimal pilot control power for each UE.

## Simulation results and discussions

In this section, the performance of uplink cell-free massive MIMO systems is evaluated by taking into account the impact of the available pilots $\tau$, the number of *M* APs, the number of *K* UEs, and the number of $N_{r}$ antennas which are equipped for each AP in the cell-free network. The performance metric in this paper is the total uplink net throughput, by taking into account the channel estimation overhead, which is defined as $B \times \left( \frac{1-\tau /\tau _{c}}{2} \sum _{k=1}^{K}\log _{2} (1 + \text {SINR}_{k})\right)$, where $B = 20$ MHz, and $\tau _{c}= 200$. In addition, ${\rho }_{p}$, and ${\rho }_{u}$ are both assumed to be 100 mW. *M* APs and *K* UEs are randomly distributed in the square area $D \times D$ $\text {km}^{2}$, where $D = 1000$ m, and the wrapped around technique is utilized to simulate a network in order to emulate the condition of being without boundaries. The large scale fading coefficients $\beta _{k,m}$ is expressed as

18 $$\begin{aligned} \beta _{k,m} = \text {PL}_{k,m} \left( 10 \frac{\sigma _{sh}\chi _{k,m} }{10}\right) , \end{aligned}$$

where $\text {PL}_{k,m}$ gives the pathloss, and $10 \frac{\sigma _{sh}\chi _{k,m} }{10}$ shows the shadow fading with standard deviation $\sigma _{sh}\chi _{k,m}$ and $\chi _{k,m} \sim {\mathscr {C}} {\mathscr {N}} (0, 1)$. Thus, three slope pathloss models are utilized in which the pathloss exponent is 3.5 when the distance between the $k_\text {th}$ UE and $m_\text {th}$ AP is denoted by $d_{k,m}$ and is larger than $d_{1}$, the pathloss exponent is 2 when ${d}_{0} < \text {d}_{k,m} \le {d}_{1}$ and 0 when ${d}_{k,m} \le {d}_{0}$, where ${d}_{0} = 10$ m and ${d}_{1} = 50$ m^4^. Therefore, the pathloss models are given as^23^

19 $$\begin{aligned} \text {PL}_{k,m} [\text {dB}] = \left\{ \begin{array}{ll} -\text {L} - 35 \log _{10}(d_{k,m}), &{}\quad \text {if } \ \text {d}_{k,m} > {d}_{1} \\ -\text {L} - 15 \log _{10}(d_{1}) -20\log _{10}({d}_{k,m}), &{}\quad \text {if } \ {d}_{0} < \text {d}_{k,m} \le {d}_{1}\\ -\text {L} - 15 \log _{10}(d_{1}) -20\log _{10}(\text {d}_{0}), &{}\quad \text {if } \ {d}_{k,m} \le {d}_{0} \end{array}\right. \end{aligned}$$

where $\text {L} = 46.3 + 33.9\log _{10}(f) - 13.82 \log _{10}(h_{AP}) - (1.1\log _{10}(f) - 0.7)h_{UE} + (1.56\log _{10}(f) - 0.8$, where *f* denotes the carrier frequency in (GHz), which is equal to 1.9 GHz. $h_{AP} = 15$ m and $h_{UE} = 1.65$ m represents the antenna height of the $m_\text {th}$ AP and the $k_\text {th}$ UE, respectively. Most earlier studies of the shadowing correlation model assumed that the shadowing coefficients are uncorrelated, however in actuality, the transmitter and receiver may be surrounded by similar obstacles. The shadowing coefficients are therefore correlated, which may have an impact on the system’s performance. Therefore, the shadowing and correlation models are described in^4^, (54)–(55). All results are obtained by using Mont Carlo simulation whereby new APs and UEs locations are randomly located in each iteration. Finally, the parameters of the proposed scheme of the pilot assignment based on the integration between the Hungarian scheme and the GA have been set as $\Gamma = 200$, the mutation probability = 0.8, and the percentage of the crossover probability is $80\%$.

**Figure 3:**
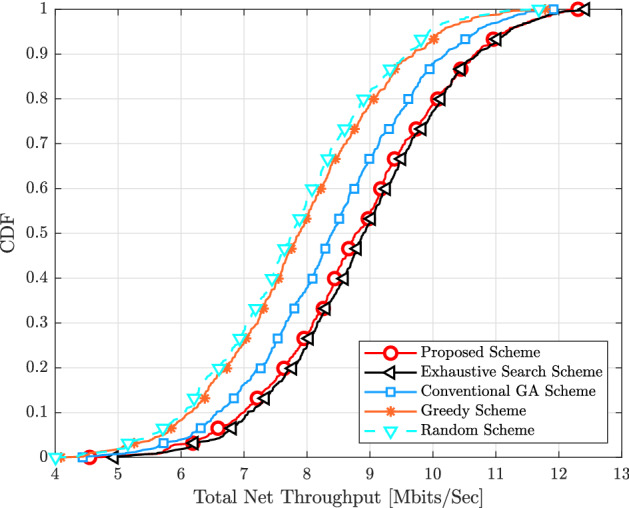
CDF of the total uplink net throughput for different pilot assignment schemes with $M = 40$, $K = 10$, $N_{r} = 1$, $\eta _{k} = 1$ and $\tau = 2$.

Figure 3 shows the cumulative distribution function (CDF) of the total uplink throughput for different pilot assignment schemes when $M = 40$, $K = 10$, $N_{r} = 1, \eta _{k} = 1$, and $\tau = 2$. The proposed scheme based on the integration between the Hungarian scheme and the GA is compared to the exhaustive search scheme in which all possible ($\tau ^{K}$) pilot sequences are evaluated and the pilot sequence that can provide the maximum total net throughput will be taken, the conventional GA, the greedy and random schemes. It is obvious that the proposed scheme can achieve almost the same as the exhaustive search scheme. This is because the proposed scheme uses the Hungarian algorithm to provide the initial population for the GA in order to search for the nearby the optimal solution rather than using random initial population as the conventional GA does. In addition, the proposed scheme can overcome other schemes with respect to both $95\%-\text {likely}$ and median of the total uplink net throughput.

**Figure 4:**
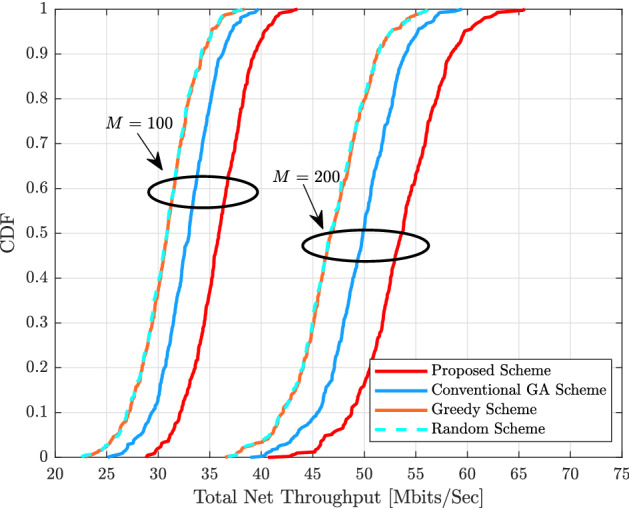
CDF of the total uplink net throughput for different pilot assignment schemes with $K = 40$, $N_{r} = 1$, $\eta _{k} = 1$, and $\tau = 5$.

Figure 4 illustrates that the CDF of the total uplink net throughput with two cases of *M* APs, as $M = 100$ and $M = 200$, with $K = 40$, $N_{r} = 1$, $\eta _{k} = 1$ and $\tau = 5$. It can be seen that the proposed pilot assignment scheme can overcome other schemes in both cases of *M*. It is also noted that the total uplink net throughput increases as *M* increases. However, there are no improvements in the gaps of both $95\%-\text {likely}$ and median due to increasing the number of APs in the cell-free network. This is because $\tau$ plays a vital role in the system performance and it is less associated with *K* UEs. For example, when $M = 200$, the proposed scheme can achieve 48.654 (Mbits/s) $95\%-\text {likely}$ total uplink net throughput compared to 45.30 (Mbits/s), 42.001 (Mbits/s), 41.8 (Mbits/s) for the conventional GA, greedy scheme, and random scheme, respectively.

**Figure 5:**
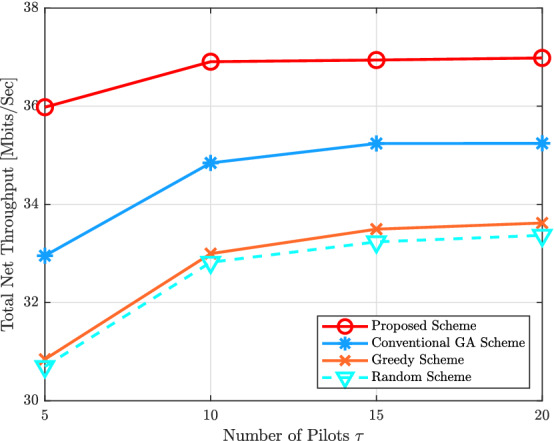
Total uplink net throughput versus various $\tau$ available pilots with $M = 100$, $K = 40$, $\eta _{k} = 1$, and $N_{r} = 1$.

Figure 5 demonstrates the total uplink net throughput versus various available pilots $\tau$, when $M = 100$, $K = 40$, $\eta _{k} = 1$, and $N_{r} = 1$. It can be seen that the total uplink net throughput slowly increases as the number of available pilots $\tau$ increases because when $\tau$ becomes large, the time for uplink data transmission per coherence interval becomes small. On the other hand, when $\tau$ is small, the accuracy of the estimated channel decreases and this will affect on the system performance because the strong effect of the pilot contamination phenomenon. In addition, the gap between the proposed scheme and other schemes in this work increases when $\tau$ decreases. It is obvious that the proposed scheme of pilot assignment can mitigate the pilot contamination phenomenon in the uplink cell-free massive MIMO systems. For example, the total uplink net throughput of the cell-free massive MIMO network with $\tau = 10$ improves by $5.75\%$, $11.2\%$, and $12\%$ comparing with the conventional GA, the greedy scheme, and the random scheme, respectively.

**Figure 6:**
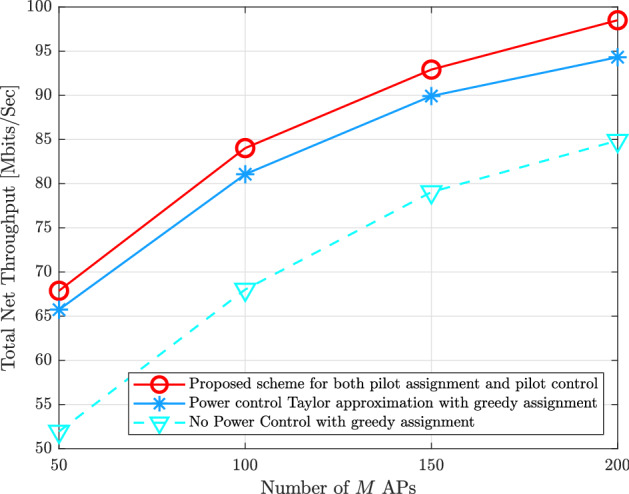
Total uplink net throughput for the proposed schemes of both pilot assignment as well as pilot power control compared to the state-of-the-art schemes versus various *M* APs with $K = 40$, $\tau = 5$, $\eta _{k} = 1$, and $N_{r} = 16$.

Figure 6 provides the total uplink net throughput of the proposed scheme for both pilot assignment, and the pilot power control design compared with the state-of-the-are schemes which are the pilot power control design using second-order Taylor approximation with greedy pilot assignment^9^, and the greedy pilot assignment when all UEs in the cell-free network transmit their pilot signals with full pilot power^4^. Regarding the pilot power control design using second-order Taylor approximation, It has presented a min-max optimization problem that aims to reduce the largest of all UEs’ normalized mean-squared channel estimation errors. This proposed optimization problem is non-convex. Then, it was changed to the second-order Taylor approximation. It is obvious that the proposed scheme in this paper offers around $5\%$, and $18\%$ improvement in the total uplink net throughput compared with the pilot power control design^9^ and full pilot power transmission during the training phase in^4^, respectively. In addition, it can be seen that small number of APs with small available pilots $\tau$ increase the pilot contamination effect and the proposed scheme can mitigate the effect of pilot contamination.

**Figure 7:**
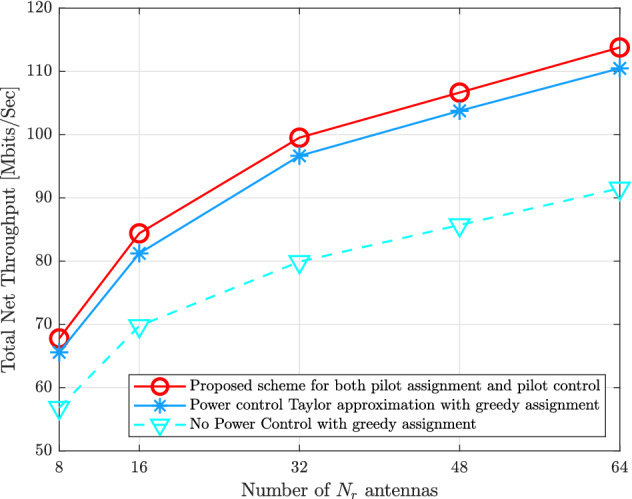
Total uplink net throughput for the proposed schemes of both pilot assignment as well as pilot power control compared to the state-of-the-art schemes versus various $N_{r}$ receive antennas with $M = 100$, $K = 40$, and $\tau = 5$.

Figure 7 shows the impact of the $N_{r}$ antennas on the total uplink net throughput of the proposed schemes for both pilot assignment and power control compared to^4^, and^9^. As expected, as the number of $N_{r}$ increases, the uplink net throughput of all schemes increase. However, the proposed schemes in this paper can offer around $3\%$, and $22\%$ improvement compared to the mentioned schemes. This is because the greedy pilot assignment of other schemes cannot obtain the optimal pilot sequences for *K* UE, while the proposed pilot assignment can achieve near to the optimal results as mentioned previously.

**Figure 8:**
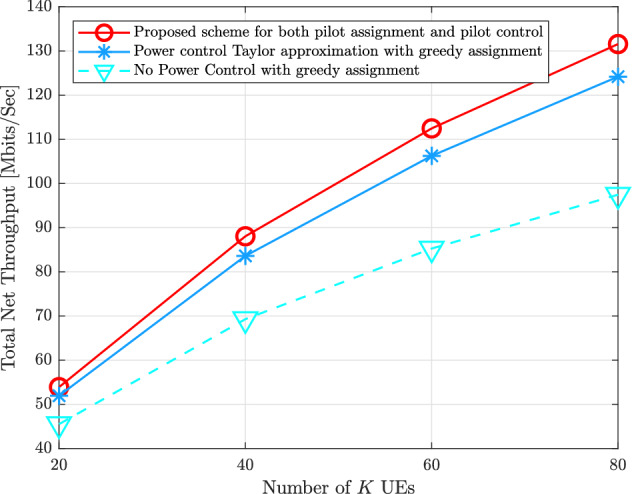
Total uplink net throughput for the proposed schemes of both pilot assignment as well as pilot power control compared to the state-of-the-art schemes versus various *K* UEs with $M = 100$, $N_{r} = 16$, and $\tau = 5$.

Figure 8 indicates the total uplink net throughput with $K = \{20,40,60,80\}$ with $N_{r} = 16$, $M = 100$, and $\tau = 5$. It is obvious that the uplink net throughput increases as *K* increases. This is because the inter-user interference cannot affect on the spectral efficiency of the uplink systems. The proposed schemes in this paper can overcome the mentioned schemes. For example, when $K = 60$, the proposed schemes to mitigate the pilot contamination can attain 112.464 [Mbits/s], the pilot power control with greedy assignment achieves 106.254 [Mbits/s], and the greedy pilot assignment without pilot power control achieves 85.2676 [Mbits/s].

**Figure 9:**
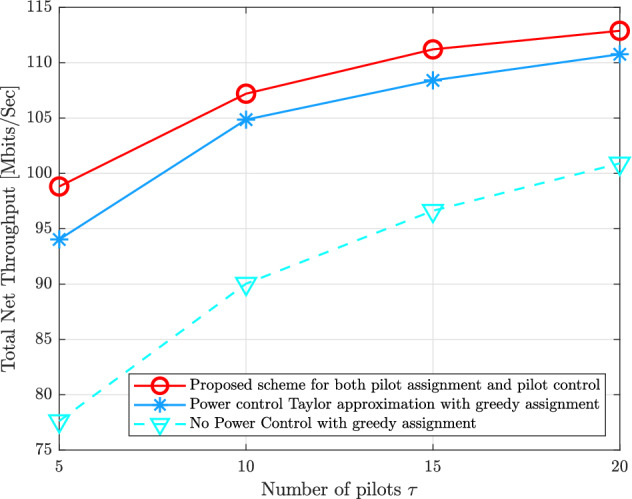
Total uplink net throughput for the proposed schemes of both pilot assignment as well as pilot power control compared to the state-of-the-art schemes versus various $\tau$ available pilots with $M = 100$, $N_{r} = 16$, and $K = 40$.

Figure 9 demonstrates the impact of several numbers of the available pilots $\tau$ on the system performance in terms of the total uplink net throughput. It can be seen that the proposed schemes for both pilot assignment as well as the pilot power control design can attain better performance compared to other schemes. In addition, it is obvious that taking into consideration both of pilot assignment and pilot power control coefficients can strongly enhance the system performance by mitigating the pilot contamination effect.

## Complexity analysis

**Figure 10:**
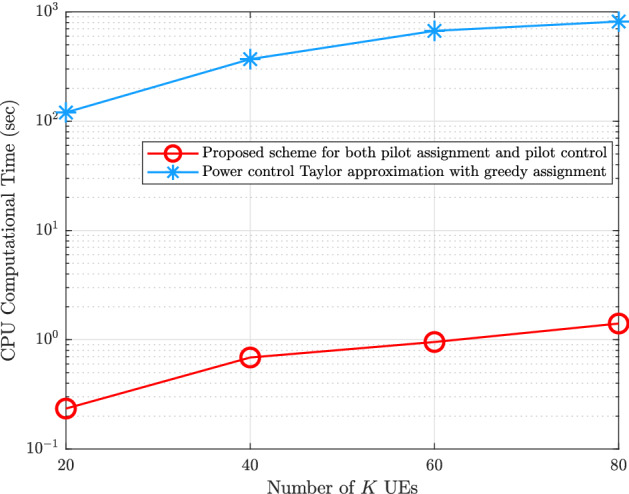
CPU computational time comparison of our proposed schemes of mitigation the pilot contamination against power control design using a Taylor approximation with greedy assignment scheme for increasing number of *K* UEs.

The complexity analysis of the proposed scheme based on the integration between the Hungarian method and the GA is ${\mathscr {O}}(\Gamma {\mathscr {P}}K)$, where ${\mathscr {P}}$ is the population size and it is equal to *K*, while the complexity analysis of the conventional GA differs from the proposed scheme by the population size, which is $\tau ^{K}$^14,15^. In addition, the complexity analysis of the benchmark greedy scheme, the random scheme, and the exhaustive search scheme are ${\mathscr {O}}(KM)$, ${\mathscr {O}}(K)$, and ${\mathscr {O}}(\tau ^{K})$, respectively. On the other side, the CPU computational time in seconds is proposed to analyse the complexity of the proposed schemes for both pilot assignment and the pilot power control compared to the pilot power control based on using second-order Taylor approximation with greedy pilot assignment as shown in Fig. 10. It has presented a min-max optimization problem that aims to reduce the largest of all UEs’ normalized mean-squared channel estimation errors. This proposed optimization problem is non-convex. Then, it was changed to the second-order Taylor approximation. This scheme of pilot power control and greedy pilot assignment is used to compare with our proposed pilot assignment and pilot power control. Therefore, the proposed methodology and the second-order Taylor approximation pilot power control scheme with greedy pilot assignment seek to mitigate the pilot contamination phenomenon. In contrast, our proposed method is more scalable when many UEs exist in the coverage area and has lower computational complexity.

## Conclusion

In this paper, we proposed two novel schemes based on the matching technique for mitigating the pilot contamination in the uplink cell-free massive MIMO systems. In the first scheme, we proposed integrating the Hungarian method and the GA to assign the pilot sequences to the *K* UEs in the coverage area. We formulated an assignment optimization problem to select the best possible pilot sequences to be an input for the GA to iteratively obtain the optimal pilot sequences that can maximize the uplink SE. In the second scheme to alleviate the pilot contamination effect, we proposed a novel pilot power control design based on a matching technique between random pilot power and its corresponding channel estimation accuracy for each UE. Therefore, we formulated a minimum-weighted assignment optimization problem and solved it using the Hungarian algorithm. We also investigated our proposed schemes’ total uplink net throughput. We compared the findings to state-of-the-art strategies for pilot assignment schemes and pilot power control design by considering the impact of the number of *M* APs, *K* UEs, $N_{r}$ antennas, and $\tau$ available pilots. Our proposed methodology has a significantly lower computational complexity with higher total uplink net throughput. It would be possible for future work to utilize the proposed schemes based on the matching theory for mitigating the pilot contamination phenomenon in the cell-free millimeter wave (mm-Wave) massive MIMO systems, which can be also incorporated with antenna selection or radio frequency (RF) chain activation to enhance the channel estimation accuracy and reduce the power consumption, respectively.

## Data Availability

This work is based on MATLAB simulation. Thus, all codes can be provided by the the authors on reasonable request.
